# Tumor Metabolism Is Affected by Obesity in Preclinical Models of Triple-Negative Breast Cancer

**DOI:** 10.3390/cancers14030562

**Published:** 2022-01-23

**Authors:** Caner Yelek, Lionel Mignion, Adrien Paquot, Caroline Bouzin, Cyril Corbet, Giulio G. Muccioli, Patrice D. Cani, Bénédicte F. Jordan

**Affiliations:** 1 Biomedical Magnetic Resonance Research Group, Louvain Drug Research Institute, Université Catholique de Louvain, UCLouvain, B-1200 Brussels, Belgium; caner.yelek@uclouvain.be (C.Y.); lionel.mignion@uclouvain.be (L.M.); 2 Metabolism and Nutrition Research Group, Louvain Drug Research Institute, Walloon Excellence in Life Sciences and BIOtechnology (WELBIO), Université Catholique de Louvain, UCLouvain, B-1200 Brussels, Belgium; patrice.cani@uclouvain.be; 3 Bioanalysis and Pharmacology of Bioactive Lipids Research Group, Louvain Drug Research Institute, Université Catholique de Louvain, UCLouvain, B-1200 Brussels, Belgium; adrien.paquot@uclouvain.be (A.P.); giulio.muccioli@uclouvain.be (G.G.M.); 4 IREC Imaging Platform, Institut de Recherche Expérimentale et Clinique, Université Catholique de Louvain, UCLouvain, B-1200 Brussels, Belgium; caroline.bouzin@uclouvain.be; 5 Pole of Pharmacology and Therapeutics, Institut de Recherche Expérimentale et Clinique, Université Catholique de Louvain, UCLouvain, B-1200 Brussels, Belgium; cyril.corbet@uclouvain.be

**Keywords:** obesity, breast cancer, metabolism, dynamic nuclear polarization, NMR spectroscopy, TCA metabolites: carbon 13, metabolic flux

## Abstract

**Simple Summary:**

Obesity promotes both development and progression of breast cancer. As a disease, obesity is followed by hyperglycemia, hyperinsulinemia, and hyperlipidemia. The impact of obesity, accumulation of fat depots, and related markers on the metabolism of tumors still remains poorly understood. The aim of this study is to characterize the putative differences in the metabolism of tumors from obese and lean mice. The findings reported here could help tailor personalized treatments targeting tumor metabolism in obese cancer patients by identifying the metabolic preferences of these tumors.

**Abstract:**

Obesity is characterized by an excessive fat mass accumulation associated with multiple disorders, including impaired glucose homeostasis, altered adipokine levels, and hyperlipidemia. Despite clear associations between tumor progression and obesity, the effects of these disorders on tumor metabolism remain largely unknown. Thus, we studied the metabolic differences between tumors of obese and lean mice in murine models of triple-negative breast cancer (E0771 and PY8819). For this purpose, a real-time hyperpolarized 1-^13^C-pyruvate-to-lactate conversion was studied before and after glucose administration in fasting mice. This work was completed by U-^13^C glucose tracing experiments using nuclear magnetic resonance (NMR) spectroscopy, as well as mass spectrometry (MS). Ex vivo analyses included immunostainings of major lipid, glucose, and monocarboxylic acids transporters. On the one hand, we discovered that tumors of obese mice yield higher lactate/pyruvate ratios after glucose administration. On the other hand, we found that the same tumors produce higher levels of lactate and alanine from glucose than tumors from lean mice, while no differences on the expression of key transporters associated with glycolysis (i.e., GLUT1, MCT1, MCT4) have been observed. In conclusion, our data suggests that breast tumor metabolism is regulated by the host’s physiological status, such as obesity and diabetes.

## 1. Introduction

Among all cancer types, breast cancer (BC) is the most diagnosed cancer when both sexes are considered, with an incidence of 11.7% of all cases and 24.5% of cancers in women. It is also the most prevalent cancer-related death in women, with a mortality of 15.5% [1]. Several risk factors have been associated with BC, including, but not limited to, genetic predisposition, lifestyle, and environmental factors, such as advanced age for first pregnancy, alcohol consumption, physical inactivity, and obesity [2]. The latter is a worldwide pandemic disease defined by an excessive fat accumulation that accounts for over 650 million obese adults in 2016 (World Health Organization). Epidemiological evidence has been reported to link obesity with a higher risk of developing breast cancer, as well as adverse outcome and poorer prognosis, including bigger tumor size, shorter disease-free survival, and overall survival [3,4].

Various mechanistic hypotheses have been proposed in relation with the physiological changes induced by obesity in the adipose tissue, endocrine system, and energy homeostasis. Among the different hypotheses, we can discuss the following:

On the one hand, circulating free fatty acid (FFA) levels are elevated in obese subjects [5] with a higher release of FFA by the adipose tissue [6]. The increased levels of FFAs could also inhibit the action of insulin, thus impairing the insulin-induced inhibition of lipolysis, further increasing plasma FFA levels [7,8]. Likewise, fatty acid metabolism has been recently highlighted as a major metabolic pathway in cancer progression [9]. Furthermore, obesity-associated adipose tissue overgrowth is relevant in the context of breast cancer due to the abundance of peritumoral fat depots. Indeed, adipose tissue has been shown to support tumor growth and metastasis in several types of cancer, including ovarian, prostatic, and breast cancer [10,11]. Accordingly, peritumoral adipocytes display higher rates of lipolysis and consequently smaller lipid droplet size [12]. In vitro coculture studies have highlighted an exchange of FFAs from the adipocytes to the cancer cells with subsequent decrease in the lipid content of adipocytes and increase in the fatty acid metabolism in cancer cells, stimulating the proliferation and invasiveness of the latter [12,13,14,15].

On the other hand, adipose tissue overgrowth is characterized by adipocyte hypertrophy and hyperplasia, an increase in the size and number, respectively [16]. Accordingly, adipocyte hypertrophy is deleterious for adipose tissue function and health, with hypoxic stress occurring and leading to inflammation by the recruitment of immune cells, altered adipokine secretion, and production of pro-inflammatory cytokines, such as IL-6, TNF-α, and IL-10 [17,18]. Together with increased levels of FFA in the circulation, these phenomena promote the development of insulin resistance and are associated with high plasma levels of insulin and glucose [19]. It has also been reported that serum insulin-like growth factor-1 (IGF-1) levels are elevated in obese subjects [20]. Both insulin and IGF-1 are capable of activating the PI3K/AKT/mTOR pathway by binding to the insulin receptor, thereby promoting glycolytic activity [21].

Finally, in preclinical models of breast cancer, we and others have shown that obese, high-fat diet (HFD) fed mice exhibit bigger tumors and faster progression than lean, normal diet (ND) fed mice [22,23,24]. Although physiological alterations occurring during obesity, as listed above, are under investigation, whether the tumor metabolic phenotype is different in obese versus lean environment remains unknown. For this purpose, we have characterized, in two distinct models of breast cancer, the metabolism of tumors of lean and obese mice by performing state-of-the art (i) hyperpolarized 1-^13^C-pyruvate-to-lactate label exchange, (ii) uniformly labeled ^13^C-glucose tracing experiments by nuclear magnetic resonance (NMR) and (iii) mass spectrometry (MS). Lastly, we performed immunohistochemical (IHC) staining in order to investigate the differences in the expression patterns of main metabolic transporters, as well as environmental markers.

## 2. Materials and Methods

### 2.1. Cell Culture

E0771 and PY8119 *Mus musculus* mammary gland adenocarcinoma cell lines were acquired from American Type Cell Culture (ATCC, Manassas, VA, USA) and stored according to the supplier’s instructions. E0771 cells were maintained in Dulbecco’s Modified Eagle Medium (GIBCO, Thermo Fisher Scientific, Waltham, MA, USA) with 25 mM glucose, 4 mM glutamine, and 25 mM HEPES, supplemented with 10% heat-inactivated Fetal Bovine Serum (FBS) (Thermo Fisher Scientific, Waltham, MA, USA). PY8119 cells were maintained in culture in F-12K medium (GIBCO, Thermo Fisher Scientific, Waltham, MA, USA) with 7 mM glucose and 2 mM glutamine, supplemented with 5% heat-inactivated FBS (Thermo Fisher Scientific, Waltham, MA, USA). They were cultured in a humidified atmosphere at 37 °C and 5% CO_2_.

### 2.2. Mice

All mouse experiments were approved by the ethical committee for animal care of the Health Sector of the Université Catholique de Louvain, under the supervision of JP Dehoux (Head veterinarian), under the specific number 2018/UCL/MD/021 and performed in accordance with the guidelines of the local ethics committee and in accordance with the Belgian Law of 29 May 2013, regarding the protection of laboratory animals (agreement number LA1230467). Specific pathogen-free (SPF) certified 8-week-old female C57BL/6JRj mice (Janvier Labs, Le Genest-Saint-Isle, France) were used for the experiments. Cages were randomly assigned to experimental groups to ensure that each group was matched in terms of bodyweight at the beginning of normal diet (D10012M; Research Diets, New Brunswick, NJ, USA) and high-fat diet 60% kcal (D12492; Research Diets, New Brunswick, NJ, USA) feeding. ND is composed of 73 gr/100 gr carbohydrates, 14 gr/100 gr protein, and 4 gr/100 gr fat (rich in unsaturated lipids), whereas the macronutrient distribution in the HFD is 26 gr/100 gr carbohydrates, 26 gr/100 gr protein, and 35 gr/100 gr fat (mainly saturated fats). Bodyweight was assessed weekly. All of the experiments, including hyperpolarized ^13^C-NMR spectroscopy, as well as U-^13^C-glucose administration and glycemia acquisition, were performed on the same cohort of mice.

### 2.3. Tumor Growth and Tissue Sampling

Tumors were induced by subcutaneous injection of 1 × 10^6^ cells in the fifth mammary fat pad of C57Bl/6JRj female mice 6 weeks after the beginning of the HFD treatment. The cells were freshly passaged just before the injection and prepared as a mixture of 1:1 PBS and Matrigel (Corning, Amsterdam, The Netherlands) and injected within 30 min. Tumor size was monitored at least twice a week and measured using an electronic caliper in a simple blind manner. At the end of the experiment, mice were anesthetized with isoflurane after a fasting period of 6 h. Blood was sampled by a cardiac puncture. After blood sampling, mice were killed by cervical dislocation. Tumors were precisely dissected, cut in half, and immediately immersed in liquid nitrogen followed by storage at −80 °C for further analysis or 4% paraformaldehyde (PFA) for tissue fixation.

### 2.4. MR Experiments

MR experiments were performed on an 11.7-Tesla, 16-cm inner diameter system (Bruker, Biospec, Karlsruhe, Germany), equipped with a double tuned ^1^H-^13^C-surface coil (RAPID Biomedical, Rimpar, Germany), as previously described [25]. Mice were anesthetized by isoflurane inhalation (2.5% in air for induction and 1–2% in air for maintenance). Body temperature was maintained at 37 °C using a warm circulating water blanket and monitored using a rectal temperature probe. A pressure cushion was used to monitor breathing, allowing adaptation of anesthetic gas flow when needed. Alongside providing reference images, anatomical T2-weighted images were used to assess tumor volume. The turbo RARE sequence had the following parameters: repetition time (TR) = 2.5 s; echo time (TE) = 30 ms; averages = 2; field of view = 3 × 3 cm; 15 slices with a 1-mm thickness.

### 2.5. Hyperpolarized ^13^C-NMR Spectroscopy and Data Analysis

Hyperpolarized ^13^C-NMR data were acquired, as previously described [26]. 1-^13^C pyruvic acid (Sigma-Aldrich, Saint Louis, MO, USA) was mixed with 15 mM trityl radical OXO63 and doped with 2 mM gadolinium (Guerbet, Villepinte, France). The solution of 40 μL was hyperpolarized by an Oxford DNP Polarizer (HyperSense, Oxford, UK) for approximately 45 min at 1.4 K and 3.35 T. The polarized substrate was quickly dissolved in 3 mL of heated buffer containing 100 mg/L EDTA, 40 mM HEPES, 30 mM NaCl, 80 mM NaOH, and 30 mM of lactate. The final solution was adjusted to pH 7 and quickly injected using a catheter into the tail vein of the mice in the MRI scanner (11.7-Tesla, Bruker, Biospec, Karlsruhe, Germany). Mice were scanned using a double tuned ^1^H-^13^C-surface coil (RAPID Biomedical, Rimpar, Germany), which was designed for spectroscopy of subcutaneous tumors (i.e., tumor-shaped cavity of 12 mm in diameter). This experiment was first done on fasted mice, after a period of 30 min glucose (0.75 mg/g of mouse) was injected by i.v., and 13 min after, a second injection of hyperpolarized pyruvate was conducted. This timing was chosen according to intravenous glucose tolerance test (IVGTT) data available in the literature [27,28]. After administration of 0.2 mL of hyperpolarized pyruvate, ^13^C spectra were acquired using a single pulse sequence every 3 s for 210 s (70 repetitions), a flip angle of 10°, and an acquisition bandwidth of 50 kHz (10,000 points). Peak areas under the curve were measured for each repetition time and each time point using homemade routines in MATLAB R2018b (Mathworks, Portola Valley, CA, USA). The integrated peak intensities of hyperpolarized ^13^C-pyruvate, ^13^C-lactate, and ^13^C global signal were measured.

### 2.6. U-^13^C-Glucose Administration and Glycemia Acquisition

Mice were fasted at least 6 h before the experiment. Blood glucose levels were measured 15 min before (time point −15), just prior to intraperitoneal U-^13^C-glucose injection (2 mg/g of mouse) (time point 0), and then every 15 min until the sacrifice at the 75th min and sampling of the tumor. The timepoint was chosen according to the protocol published by Yuan and colleagues [29]. Glycemia was determined with a glucose meter (Accu Check, Roche, Switzerland) on blood samples collected from the tip of the tail vein.

### 2.7. Metabolite Extraction and ^13^C-MRS

Polar metabolites were extracted from snap-frozen tumor tissue, as previously described [29,30]. Briefly, around 100 mg of tissue was homogenized in ice-cold methanol (4 µL/mg) with the help of a mechanical lyser (TissueLyser II, Qiagen, Hilden, Germany). The homogenate was then centrifuged at 14,000 *g* at 4 °C for 20 min. All the supernatant was collected and centrifuged again with the same parameters, and debris were eliminated. After the second centrifugation, the same volume of extract for each sample was collected in glass NMR tubes. The solvent was completely removed using a vacuum concentrator. The sample was reconstituted in 600 μL sodium phosphate buffer with 10% deuterium oxide containing 0.75 wt % 3-(trimethylsilyl) propionic-2,2,3,3-d4 acid (TSP) (Sigma-Aldrich, Saint Louis, MO, USA). ^13^C NMR spectra were acquired on a 600 MHz NMR (Bruker, Biospec, Karlsruhe, Germany) equipped with a broadband cryoprobe, as described previously [31]. The acquisition time was 0.8 s with 2048 repetitions, 10 s of interpulse delay (1D sequence with inverse gated decoupling using 30° flip angle). Spectrum analysis, assignment, and quantification were performed with MestReNova software version 14.2.0-26256 (Santiago de Compostela, Spain).

### 2.8. Metabolite Extraction and Mass Spectrometry

For tricarboxylic acid metabolite isotope quantification, we used a derivatization method followed by UPLC-MS analysis using a LTQ-Orbitrap XL mass spectrometer [32]. Briefly, tumor samples were homogenized before adding acetonitrile and valproic acid (internal standard). After 1h at −20 °C, samples were centrifuged and supernatant recovered. The derivatization reaction was then conducted using 3-nitrophenylhydrazine, *N*′-ethylcarbodiimide hydrochloride, and pyridine. Following the reaction, the derivatized products were recovered by liquid-liquid extraction and the samples resuspended in methanol before injection on the LC-MS system. Separation was conducted on a hypersil gold PFP column (Thermo Fisher Scientific, Waltham, MA, USA) maintained at 40 °C using a gradient between water-acetonitrile (A) and acetonitrile (B), both acidified with acetic acid. Tricarboxylic acid metabolites (as well as their isotopic forms) were analyzed using the LTQ-Orbitrap XL analyzer (Thermo Fisher Scientific, Waltham, MA, USA) equipped with an APCI probe operated in positive mode. For each analyte, the signal (AUC) was normalized with the signal of the internal standard and further normalized with the sample weight. Based on glucose metabolism, for pyruvate and lactate we analyzed M+1, M+2, and M+3 isotopes, and for the other metabolites we analyzed the M+1, M+2, M+3, and M+4 isotopes.

### 2.9. Immunohistochemical Analyses

Tumors were fixed in 4% paraformaldehyde for 48 h at 4 °C before processing for paraffin embedding. All IHC were performed in technical duplicates on 5-µm tumor sections. After being submitted to antigen retrieval with either citrate buffer at pH 5.7 or Tris-EDTA buffer at pH 9, depending on the antibody manufacturer’s instructions, sections were incubated in BSA 5% in TBS/Triton 0.05% to block non-specific binding, then overnight at 4 °C with primary antibodies for CAIX (Novus Bio, NB100-417), CD31 (Cell Signaling, 77699), CD36 (Sigma-Aldrich, HPA002018), CPT1a (Abcam, ab234111), (GLUT1 (Proteintech, 21829-1-AP), MCT1 (Proteintech, 20139-1-AP), and MCT4 (Sigma-Aldrich, HPA021451). These primary antibodies were revealed with Envision peroxidase-conjugated anti-rabbit secondary polymer antibody and DAB chromogen (Dako-Agilent, Santa Clara, CA, USA). Sections were eventually counterstained with hematoxylin (Dako-Agilent). Stained slides were then digitalized using a SCN400 slide scanner (Leica Biosystems, Buffalo Grove, IL, USA) at 240× magnification and subjected to blind analysis to obtain the percentage of stained tissue using Visiopharm software.

## 3. Results

### 3.1. Obese and Glucose Intolerant Mice Exhibit Bigger Breast Tumors Than Lean Mice

**Figure 1:**
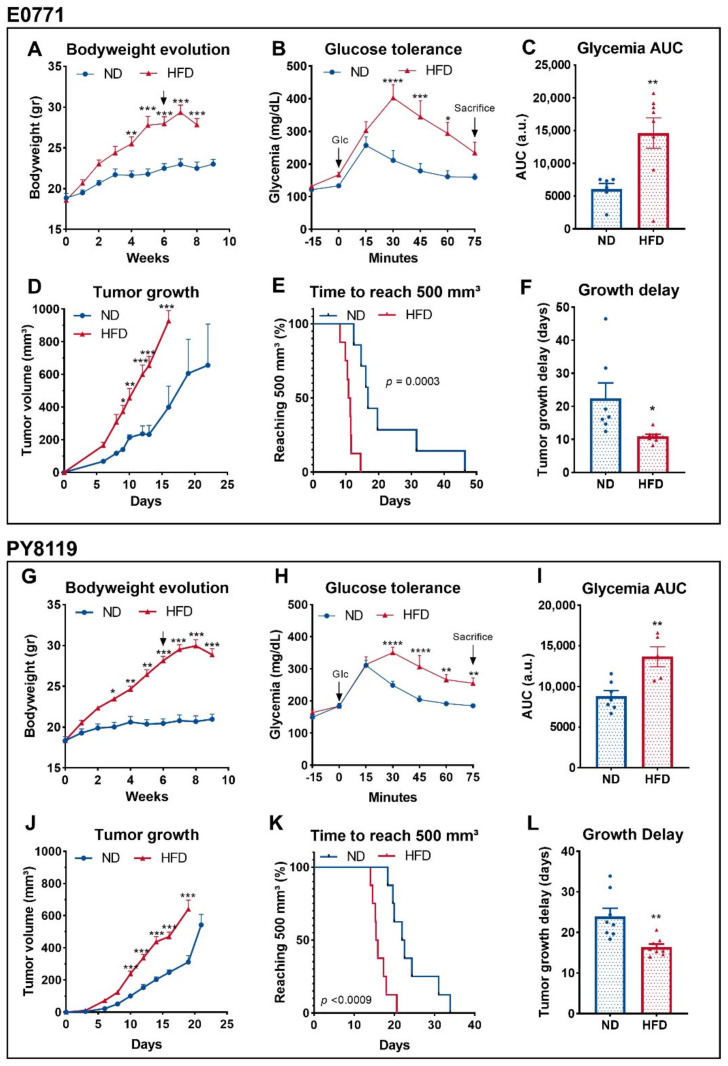
Obese and glucose intolerant mice exhibit bigger breast tumors than lean mice. (**A**–**F**): E0771 cell line; sample size: *n* = 7 for ND except for (**C**) *n* = 6 and *n* = 8 for HFD. (**G**–**J**): PY8119 cell line; sample size *n* = 8 for ND and *n* = 8 for HFD except (**I**) *n* = 7 and *n* = 5, respectively. (**A**,**G**) Bodyweight evolution under ND and HFD. Arrows indicate tumor growth’s start. (**B**,**H**) Glycemia levels measured after intraperitoneal U-^13^C glucose (2 mg/g) injection. (**C**,**I**) Area under the curve calculated from (**B**,**H**). (**D**,**J**) Tumor growth evolution. (**E**,**K**) Survival curve calculated as the time for the tumor to reach 500 mm^3^. (**F**,**L**) Tumor growth delays calculated as the amount of time needed to reach 500 mm^3^. Statistical analyses: (**A**,**B**,**D**,**G**,**H**,**J**) two-way ANOVA followed by Sidak’s multiple comparison test; * *p* < 0.05; ** *p* < 0.01; *** *p* < 0.001.; **** *p* < 0.0001. (**C**,**F**,**I**,**L**) Unpaired *t*-test; * *p* < 0.05; ** *p* < 0.01.

We and others have shown that HFD-induced obesity promotes tumor growth in mouse models of breast cancer [22,23,24]. As a condition, obesity is characterized by a cluster of several metabolic disorders [33] capable of affecting tumor metabolism [21]. Thus, we compared in vivo the tumor metabolism of two murine triple-negative breast cancer cell lines in lean and obese mice. The two models, E0771 and PY8119 cells lines, have been described as sensitive to obesity-driven tumor growth [23,34]. First, we replicated the previously described phenotype in mice developing E0771 (Figure 1A–F) or PY8119 (Figure 1G–L) tumors. As expected, HFD feeding significantly increases bodyweight (Figure 1A) and induces glucose intolerance, as shown by the higher levels of glycemia in response to intraperitoneal glucose load as compared to ND-fed mice (Figure 1B,C). Accordingly, obese mice developed bigger tumors than their lean counterparts in the same period (Figure 1D) while reaching 500 mm^3^ (biggest volume attained by the smaller tumors) in shorter delays (Figure 1E,F). Consistently, similar results regarding the bodyweight (Figure 1G) and the glucose tolerance of the mice (Figure 1H,I), as well as tumor growth (Figure 1J–L), have been obtained in mice harboring PY8119 tumors.

### 3.2. Breast Tumors of Obese Mice Display Higher Lactate over Pyruvate Ratio Than Tumors of Lean Mice

**Figure 2:**
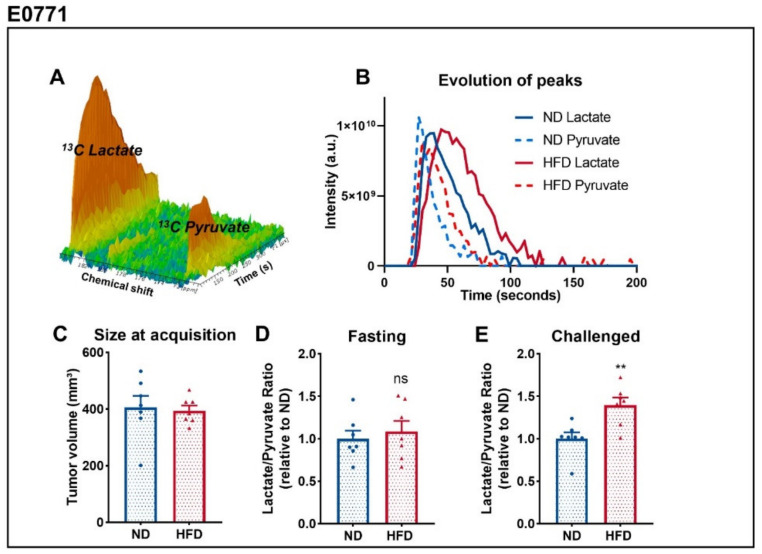
Breast tumors of obese mice display higher lac/pyr ratio than tumors of lean mice. (**A**) Representative MRS spectrum after injection of hyperpolarized 1-^13^C pyruvate acquired in vivo. (**B**) Representative evolution of ^13^C pyruvate and ^13^C lactate peaks over the time, from which the area under the curve and ratios are calculated. (**C**) Tumor size at the time of HP 1-^13^Cpyruvate-to-lactate spectra acquisition of E0771 tumors measured by T2-weighted MRI images. (**D**) Calculated Lac/Pyr ratio in fasted mice and (**E**) mice challenged with glucose. Sample size: *n* = 7 for ND and *n* = 7 for HFD, failure to acquire spectrum from a mouse from HFD. Statistical analysis: unpaired *t*-test; ns *p* > 0.05; ** *p* < 0.01.

In order to determine metabolic differences between tumors of lean and obese mice, we consecutively performed two ^13^C metabolic flux studies. For that purpose, we acquired ^13^C-MRI spectra in vivo (Figure 2A) when tumors reached a volume of 400 mm^3^ (Figure 2C) and calculated the ratio of the area under the curve of the peak of lactate and pyruvate (lac/pyr) (Figure 2B). To do so, we monitored, in vivo, the real-time label exchange of 1-^13^C pyruvate and ^13^C lactate after intravenous injection of the hyperpolarized pyruvate before and after glucose administration in fasted mice [35,36]. This experiment was conducted only on mice bearing E0771 tumors due to high lethality and low signal-to-noise ratio when done on mice bearing PY8119 tumors. In a fasting state, the lac/pyr ratio was similar between both groups (Figure 2D), indicating that the glycolytic activity in these tumors is comparable. However, when we administered glucose to mice, tumors from the HFD-fed group displayed higher lac/pyr ratios than tumors of the ND-fed group (Figure 2E). This display in lac/pyr ratio appears to be due to both an increase of the ratio between fasting and challenged mice in tumors of obese mice, as well as a decrease of the ratio in tumors of lean mice, although only the latter is statistically significant (Figure S1). These data point out that tumor response to glucose is affected by obesity, with tumors of obese mice increasing their glycolytic activity when compared to tumors of lean mice.

### 3.3. Breast Tumors of Obese Mice Produce More Lactate Than Tumors of Lean Mice

**Figure 3:**
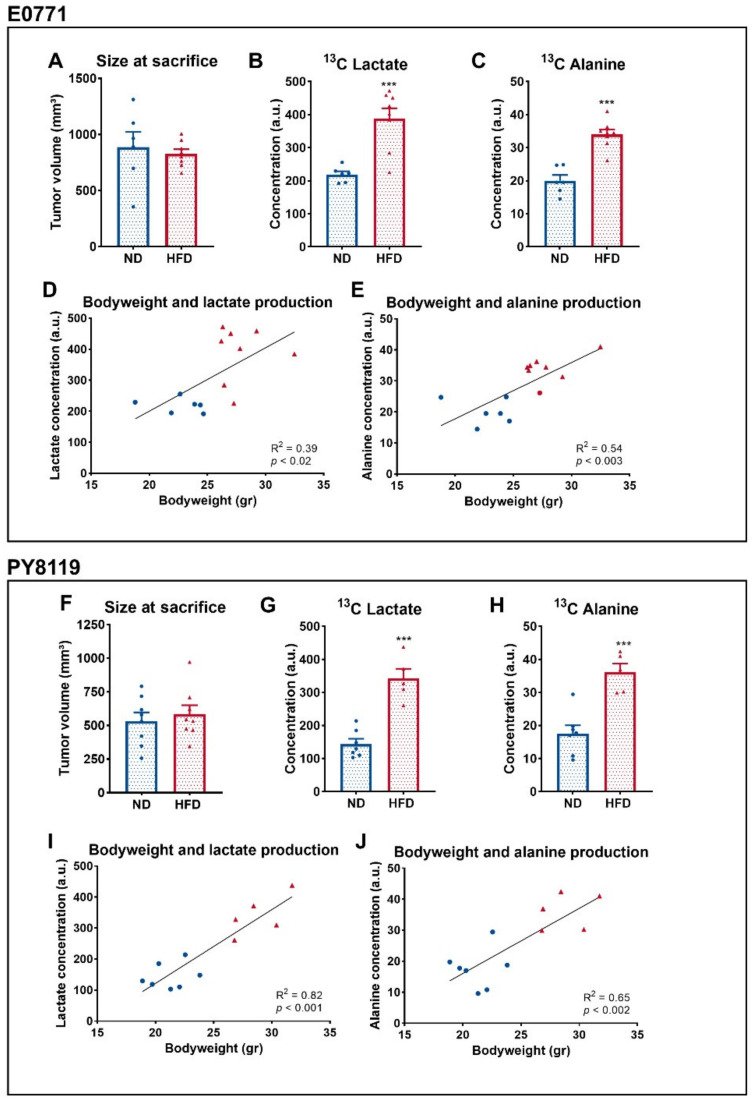
Breast tumors of obese mice produce more lactate than tumors of lean mice. (**A**–**E**): E0771 cell line; sample size: *n* = 6 for ND and *n* = 8 for HFD. (**F**–**J**): PY8119 cell line; sample size: *n* = 7 for ND and *n* = 5 for HFD. (**A**,**F**) Tumor size at the time of the necropsy. (**B**,**G**) ^13^C lactate and (**C**,**H**) ^13^C alanine concentrations from tumor extracts. (**D**,**I**) Correlation curve between the bodyweight of the mice and tumor ^13^C lactate (**E**) and ^13^C alanine (**F**,**J**) production. Statistical analysis: unpaired *t*-test; *** *p* < 0.001.

A total of 3 days after the previous experiment, using the same cohort, we fasted the mice and delivered U-^13^C_6_ glucose by intraperitoneal injection prior to the necropsy and investigated ex vivo the metabolites coming from the latter [29]. Mean tumor sizes were comparable between groups at the time of the sacrifice (Figure 3A,F). Two metabolites, lactate (Figure 3B) and alanine (Figure 3C), were significantly different between both groups and measured by NMR spectroscopy. The production of both metabolites from U-^13^C_6_ glucose was significantly increased in both the tumors of HFD-fed mice in the E0771 model (Figure 3B,C) and in the PY8119 model (Figure 3G,H). We observed a correlation between the bodyweight of the mice and the amount of lactate and alanine produced from glucose in E0771 (Figure 3D,E) and PY8119 tumors (Figure 3I,J). Altogether, these results suggest that the tumors of obese mice respond to glucose administration by an increase in the glycolytic activity, correlating with the bodyweight of the mice.

### 3.4. The Global Flux of Glucose Metabolism Is Increased in Breast Tumors of HFD-Fed Mice

**Figure 4:**
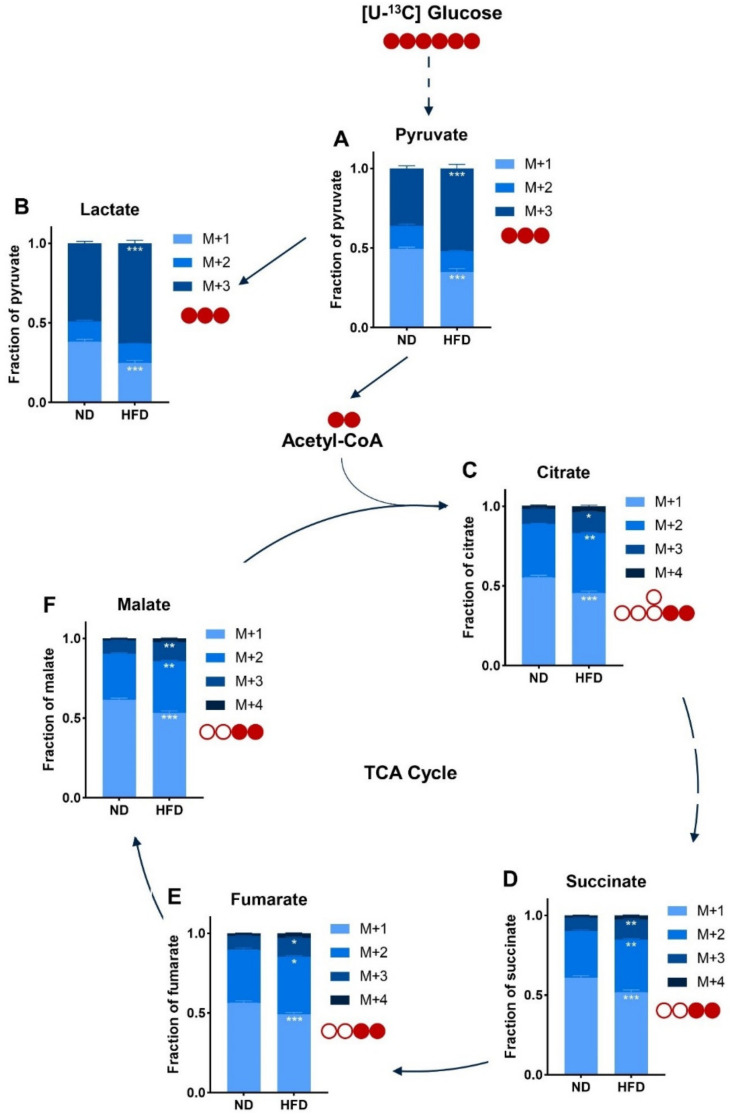
The overall flux of glucose metabolism is increased in (E0771) breast tumors of HFD-fed mice. Relative abundance of intracellular metabolites arising from U-^13^C glucose injected before the necropsy as measured by mass spectrometry. (**A**) Pyruvate; (**B**) Lactate; (**C**) Citrate; (**D**) Succinate; (**E**) Fumarate; (**F**) Malate. Sample size: *n* = 6 for ND and *n* = 8 for HFD. Statistical analyses: Two-way ANOVA followed by Sidak’s multiple comparison test, * *p* < 0.05; ** *p* < 0.01; *** *p* < 0.001.

To go further in the characterization of glucose metabolism in breast tumors of ND-fed and HFD-fed mice, we conducted targeted metabolomics by mass spectrometry analysis of major tricarboxylic acid (TCA) cycle intermediates produced by the metabolism of U-^13^C glucose. The relative abundance of M+3 pyruvate (Figure 4A) and M+3 lactate (Figure 4B) was significantly increased in extracts from tumors of obese mice, suggesting that glucose is a greater contributor in the production of both metabolites than in tumors of lean mice. Glucose contribution is around 26% higher for lactate (Figure S2A) and 15% higher for pyruvate (Figure S2B) for tumors of HFD-fed mice. Likewise, in the HFD condition, the relative M+2 labeling of citrate (Figure 4C), succinate (Figure 4D), fumarate (Figure 4E), and malate (Figure 4F) is increased compared to the ND condition. The extent of the increase is similar between all TCA cycle metabolites (Figure S2C–F). Moreover, glucose also enriched the relative M+3 labeling of TCA cycle intermediates (Figure 4C–F), suggesting an increased pyruvate carboxylase activity. Altogether, these results suggest that in tumors of obese mice there is an increased glucose metabolism, whether it be glycolysis or glucose oxidation, albeit at a lower extent for the latter.

### 3.5. The Expression of Major Metabolic Transporters Does Not Differ Except for CPT1a in Breast Tumors of Obese and Lean Mice

**Figure 5:**
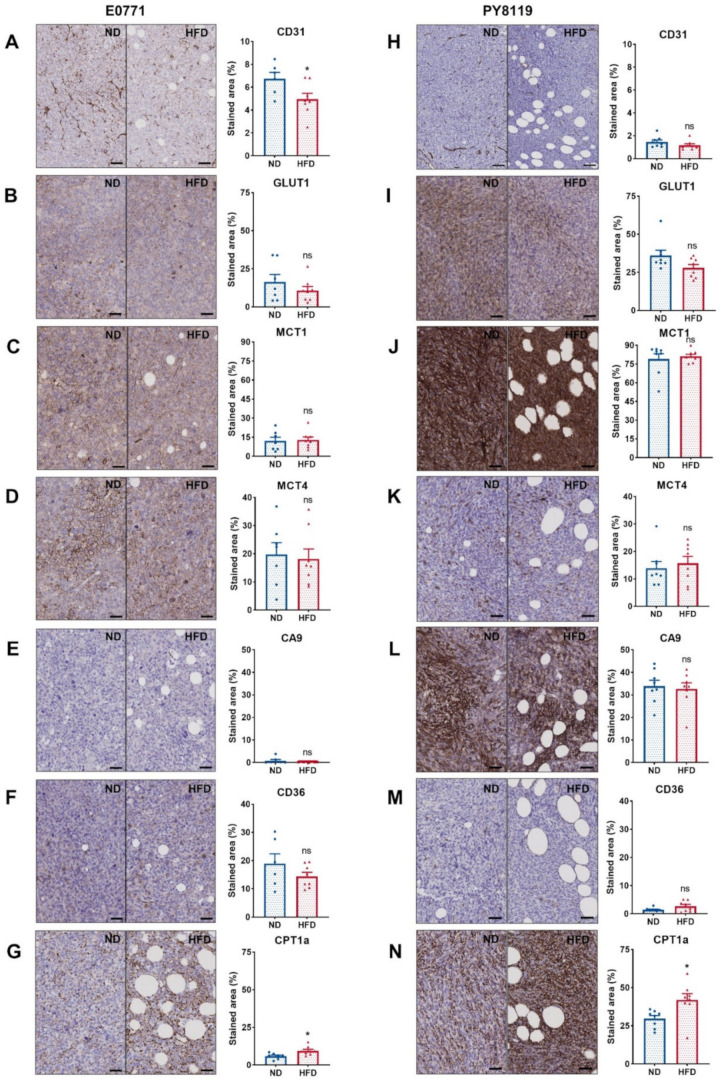
The expression of major metabolite transporters does not differ, except for CPT1a in breast tumors of obese and lean mice. (**A**–**G**): E0771 cell line; sample size: *n* = 7 for ND except (**A**) (lost sample), (**F**) (Grubb’s outlier) *n* = 6 and *n* = 8 for HFD. (**H**–**N**): PY8119 cell line; sample size: *n* = 8 for ND and *n* = 8 for HFD. (**A**,**H**) IHC staining for CD31 expression, scale bar = 100 µm. (**B**,**I**) IHC staining for GLUT1. (**C**,**J**) IHC staining for MCT1. (**D**,**K**) IHC staining for MCT4. (**E**,**L**) IHC staining for CA9. (**F**,**M**) IHC staining for CD36. (**G**,**N**) IHC staining for CPT1a. All scale bars except for CD31 = 50 µm. Statistical analyses: unpaired *t*-test; ns *p* > 0.05; * *p* < 0.05.

We carried out several immunohistochemical stainings, targeting the endothelial cell marker CD31, considered a reflect of the vascularization [37], the main transporters involved in glucose and lactate transport (GLUT1, MCT1, MCT4) [38,39], the marker of hypoxia CA9 [40], and membrane and mitochondrial fatty acid transporters CD36 [41] and CPT1a, respectively [42]. The expression of CD31 was significantly lower in E0771 tumors of HFD-fed mice than ND-fed mice (Figure 5A), while no significant difference was observed for PY8119 tumors (Figure 5H). It is worth noting that the expression of CD31, a marker of vascularization, is lower in tumors arising from the PY8119 cell line than tumors arising from the E0771 cell line (Figure 5A,H). Likewise, the expression of the hypoxic marker CA9 was remarkably higher in PY8119 tumors (Figure 5E,L). Regarding the expression of glucose and lactate transporters, no significant difference was identified between tumors of lean or obese mice for both tumor models (Figure 5B–D,I–K). However, the expression of GLUT1 and MCT1 was appreciably higher in the PY8119 model (Figure 5I,J). For the fatty acid transporters, there was no variation in the expression of CD36 between groups, albeit lower in PY8119 tumors (Figure 5F,M), while CPT1a expression was significantly increased in tumors from HFD-fed mice for both tumor models (Figure 5G,N). Interestingly, CPT1a expression is mostly localized around tumor-associated adipocytes (Figure 5G,N and Figure S3). In conclusion, the expression of major transporters, such as GLUT1, MCT1, and MCT4, involved in the metabolism of glucose and lactate, as well as the hypoxic marker CA9, was not altered by obesity in the models under study, even though tumors of HFD-fed mice showed lesser vascularization.

## 4. Discussion

In this study we characterized the growth and metabolic phenotype of two models of breast tumors developing in obese mice in comparison to tumors developing in lean mice. In accordance with previous studies [22,23,24], we have shown that obese mice develop bigger tumors than their lean counterparts in the same period, leading to shorter survival and growth delays. Moreover, HFD-fed mice developed glucose intolerance in the span of our experiment, consistent with previous reports [43]. To date, whether adipose depots associated with obesity itself or dietary lipids are the main drivers of tumor progression still remain under debate. On the one hand, preceding findings in our lab showed that HFD-fed lean mice do not display faster tumor progression [23]. On the other hand, HFD-fed mice subjected to the short-chain fatty acid acetate and presenting a normal bodyweight still exhibited enhanced tumor development [22]. In addition, recent data suggest that specific fatty acids, such as polyunsaturated FAs, might hinder tumor growth when added to the diet [44].

Upon the study of the label exchange between ^13^C Pyruvate and ^13^C Lactate, we observed that, in a fasting state, no significant difference could be observed between tumors of lean and obese mice. In opposition, when glucose was administered to these mice, there was an increase of the lactate-to-pyruvate ratio in tumors of obese mice. Moreover, ^13^C tracing experiments performed by NMR spectroscopy revealed enhancement of lactate and alanine production from ^13^C glucose in tumors of HFD-fed mice, hinting to a rise in the global flux of glucose metabolism. These results are confirmed by mass spectrometry showing that the contribution of glucose in the production of lactate, pyruvate, and, to a lesser extent, TCA cycle metabolites is greater in tumors of obese mice compared to tumors of lean mice. From a physiological point of view, the state of the mice regarding glucose homeostasis during the ^13^C tracing experiment is comparable to the label exchange study performed in a challenged state (i.e., fasting mice receiving glucose and similar glucose-induced insulin release profile) [28].

The lactate-to-pyruvate ratio reflects both MCT1 and Lactate dehydrogenase A (LDH-A) activity [45,46]; however, we have shown that MCT1 expression does not differ between the groups, suggesting that only LDH-A activity or expression is affected. Moreover, the metabolic phenotype observed in tumors from obese mice could not be due to differences on a translational level (i.e., gene expression), considering that no change in the lactate-on-pyruvate ratio was noted in a fasting state. Thus, post-translational modifications, such as phosphorylation, could be the main culprit behind this phenotype. Indeed, the PI3K-AKT signaling pathway, a well-known regulator of cancer metabolism [47], is regulated by several growth factors, such as insulin, IGF-1, and leptin, whose levels are modified in obesity [19,20,21]. In addition, even if estrogen levels were not measured because our study is focused on TNBC, we may not exclude that dysregulated levels of the former could impact tumor metabolism in other subtypes of breast cancer as estrogen is also a known activator of the PI3K-AKT signaling pathway [21]. In turn, this signaling network promotes an increased glucose utilization and glycolysis [48] by acute post-translational modifications, as well as transcriptional regulations. Correspondingly, AKT activation leads to the phosphorylation of enzymes, such as hexokinase 2 (HK2), 6-phosphofructo-2-kinase (PFKFB2), pyruvate dehydrogenase kinase 1 (PDK1), and LDH-A, thus increasing the glycolytic activity and flux [49,50,51]. Accordingly, there are no divergences in the expression of either GLUT1 or MCT4 between tumors belonging to both groups, the former being upregulated upon AKT activation [52].

Despite lower CD31 expression, which is a marker of blood vessels [37] in tumors from HFD-fed mice, as described previously [53], no significant change in the expression of CA9, which is a marker of hypoxia [40], was observed. Furthermore, CA9 levels were highly different between both cellular models, although they shared the same metabolic phenotype, suggesting that hypoxia is not a major contributor to it. This does not exclude a hypoxia-independent HIF-1ɑ activation by AKT pathway activity. In addition, CPT1a expression was increased around fat depots within the tumor, consistent with the literature [10,12,13], pointing out that local heterogeneity exists within the tumor. Still, the global metabolic phenotype is modulated by the physiological status (i.e., obesity) and should be considered while using metabolomics approaches, such as hyperpolarized substrate conversion and steady-state isotope tracing, when characterizing the metabolism.

## 5. Conclusions

In conclusion, physiological alterations occurring in obesity, such as hyperglycemia, hyperinsulinemia, and hyperlipidemia, act as environmental factors capable of regulating breast tumor metabolism. Indeed, when compared to tumors of lean mice, tumors of obese mice display higher usage and increased flux of glucose metabolism. Thus, metabolic dysregulations occurring in obesity should be taken into account while considering therapeutic strategies targeting the metabolism of the tumor.

In perspective, both hyperpolarized ^13^C pyruvate-to-^13^C lactate conversion and ^13^C glucose tracing used in this study could be applied in a clinical setting as they have been used in the context of breast and lung cancer, respectively [54,55,56], underlining that this kind of approach could help tailor personalized medicine by exploring metabolic vulnerabilities in clinical settings in complementarity to genomic approaches [57].

## Data Availability

The data presented in this study are available on request from the corresponding author.

## Supplementary Materials

The following supporting information can be downloaded at: https://www.mdpi.com/article/10.3390/cancers14030562/s1. Figure S1. Lactate-to-pyruvate ratio evolution between fasting and challenged mice. (A) Evolution of the lactate-to-pyruvate ratio from fasted state to challenged state in normal diet fed mice. (B) Evolution of the lactate-to-pyruvate ratio from fasted state to challenged state in high-fat diet fed mice. Statistical analyses: Paired *t*-test; * *p* < 0.05; Figure S2. Metabolic intermediates labeling from U-^13^C Glucose. (A) M+3 labeling for lactate. (B) M+3 labeling for pyruvate. (C) M+2 labeling for TCA cycle intermediates. Statistical analyses: Unpaired *t*-test; * *p* < 0.05, *** *p* < 0.001; Figure S3. CPT1a expression distribution. (A&D) Area covered by adipose infiltrate. (B&E) Staining index for CPT1a calculated by the intensity and the stained area for tumors of ND-fed mice. (C&F) Staining index for CPT1a calculated by the intensity and the stained area for tumors of HFD-fed mice. FA = Fatty Area. (D&I) Representative illustration of a tumor from a lean mouse. (E&J) Representative illustration of a tumor from an obese mouse. Scale bar = 400 µm. Statistical analyses: Unpaired *t*-test; * *p* < 0.05.
