# Supplementary material for: Tumor Metabolism Is Affected by Obesity in Preclinical Models of Triple-Negative Breast Cancer
Source: Cancers (Basel). 2022 Jan 23;14(3):562. doi: 10.3390/cancers14030562 (PMC8833372; doi:10.3390/cancers14030562)
Supplement: Supplementary file 1 [file cancers-14-00562-s001.zip › cancers-1537407-supple-XML.pdf]

Supplementary materials: Tumor Metabolism is Affected by Obesity in Preclinical Models of Triple-Negative Breast Cancer

Caner Yelek, Lionel Mignion, Adrien Paquot, Caroline Bouzin, Cyril Corbet, Giulio G. Muccioli, Patrice D. Cani and Bénédicte F. Jordan

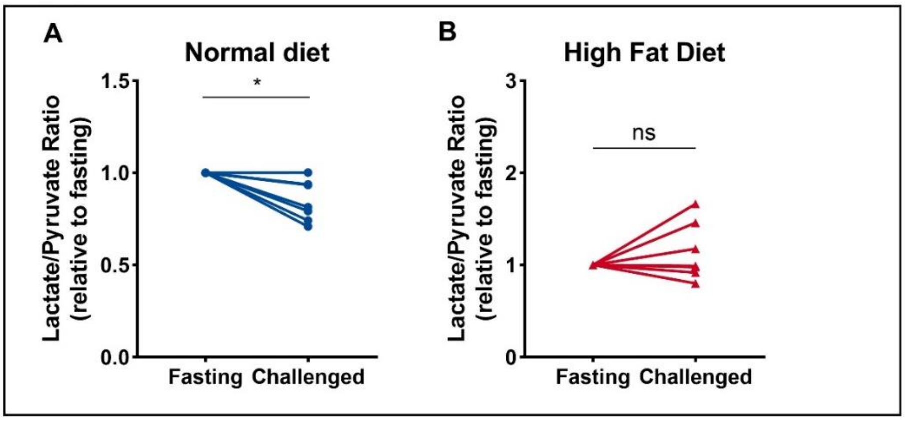

Figure S1. Lactate-to-pyruvate ratio evolution between fasting and challenged mice. (A) Evolution of the lactate-to-pyruvate ratio from fasted state to challenged state in normal diet fed mice. (B) Evolution of the lactate-to-pyruvate ratio from fasted state to challenged state in high-fat diet fed mice. Statistical analyses: Paired *t*-test; *$p < 0.05$.

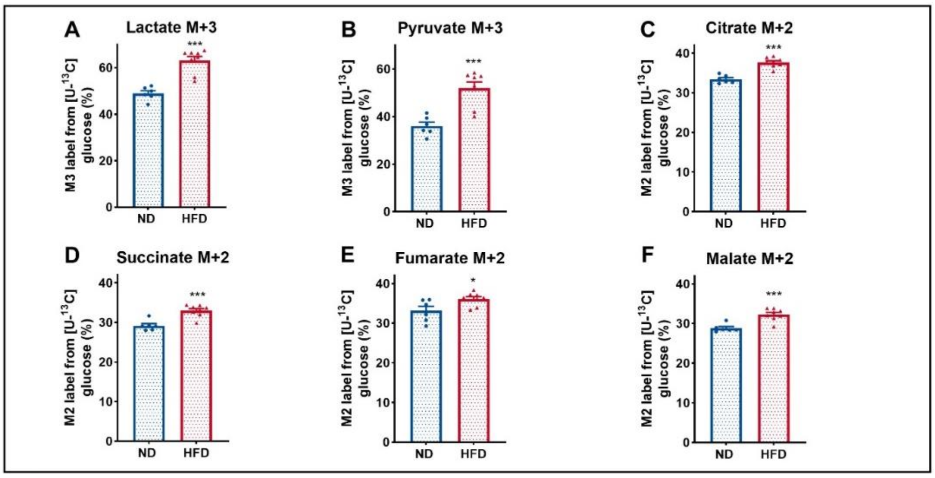

Figure S2. Metabolic intermediates labeling from U-^{13}C Glucose. (A) M+3 labeling for lactate. (B) M+3 labeling for pyruvate. (C) M+2 labeling for TCA cycle intermediates. Statistical analyses: Unpaired t-test; *$p < 0.05$, *** $p < 0.001$.

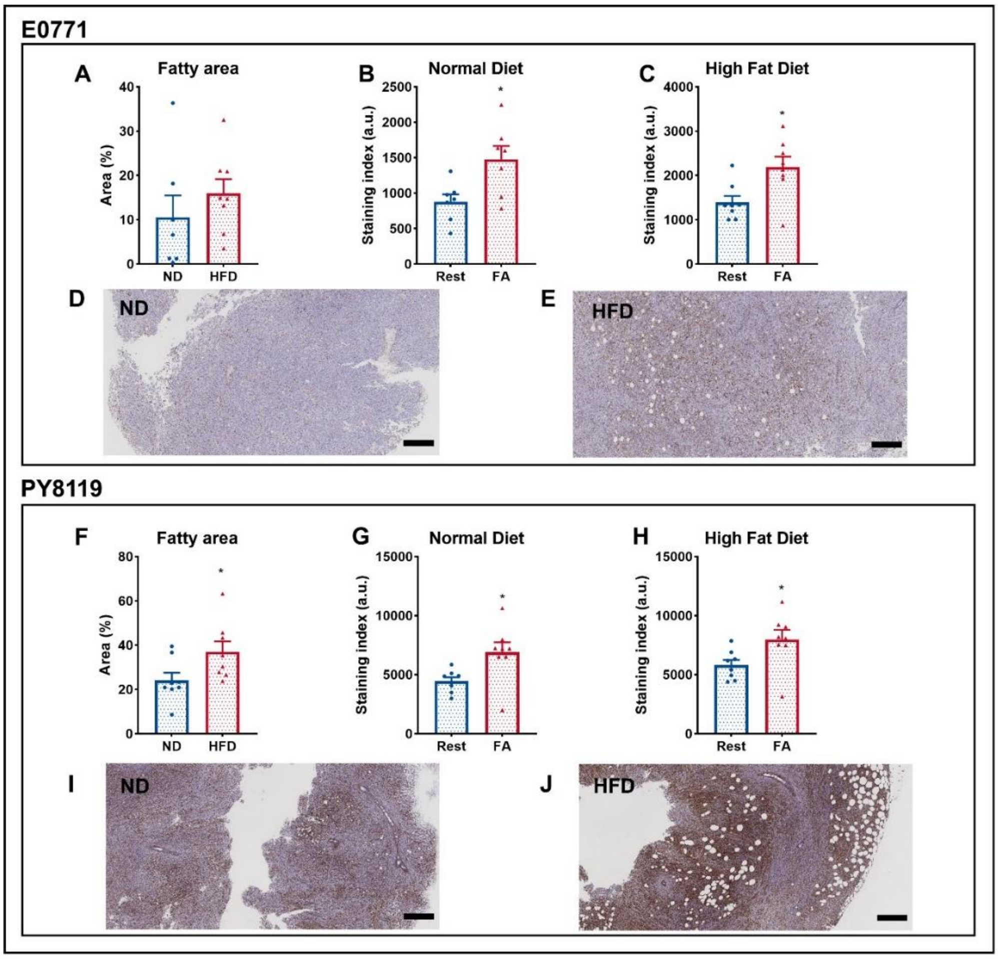

Figure S3. CPT1a expression distribution. (A&D) Area covered by adipose infiltrate. (B&E) Staining index for CPT1a calculated by the intensity and the stained area for tumors of ND-fed mice. (C&F) Staining index for CPT1a calculated by the intensity and the stained area for tumors of HFD-fed mice. FA = Fatty Area. (D & I) Representative illustration of a tumor from a lean mouse. (E& J) Representative illustration of a tumor from an obese mouse. Scale bar = 400 µm. Statistical analyses: Unpaired t-test; *$p < 0.05$.
